# Recurrent Pouch Volvulus Following Ileoanal J-Pouch Anastomosis: A Case Report

**DOI:** 10.7759/cureus.39088

**Published:** 2023-05-16

**Authors:** Mohammad Alabdulrahman, Lea Stuart, Ellie Smith, P. Ronan O'Connell

**Affiliations:** 1 School of Medicine, Royal College of Surgeons in Ireland, Dublin, IRL; 2 Surgery, University College Dublin, Dublin, IRL

**Keywords:** inflammatory bowel disease, j-pouch, ileoanal pouch anastomosis, ulcerative colitis (uc), recurrent pouch volvulus

## Abstract

For individuals suffering from severe refractory ulcerative colitis (UC) who are unresponsive to medical treatment, a total proctocolectomy and ileal pouch-anal anastomosis (IPAA) surgery is the gold-standard treatment. However, its complications include anastomotic leaks, pelvic or perianal abscesses, and rare complications such as pouch volvulus. To our knowledge, there is a scarcity of case reports on patients with, specifically, a recurrent pouch volvulus. We present a case of a 57-year-old female with refractory UC who had undergone this treatment with no initial complications; 15 years later, she presented with intermittent bouts of obstruction. An exploratory laparotomy was performed; however, no adhesions or necrosis were found. Following investigations, pouch volvulus was confirmed. She subsequently underwent four endoscopic decompressions in the same year and ultimately received an enteropexy of the pouch. The volvulus reoccurred and, ultimately, the decision was made to perform a loop ileostomy. The patient, to date, is alive and doing well with her permanent ileostomy.

## Introduction

An ileal pouch volvulus occurs when the pouch wraps around its mesenteric axis; if it is not promptly treated, obstruction and ischaemia may occur. Pouch volvulus is a rare complication of an ileal pouch-anal anastomosis (IPAA) surgery, occurring in roughly 0.18% of cases [1]. Pouch volvuli are not limited to a specific time frame postoperatively, as they may occur several weeks or even several years after the operation. Risk factors for pouch volvulus include low BMI, female gender, the orientation of the pouch, and pelvic floor dysfunction post-surgery [1]. Because pouch volvulus is a rare complication, its reoccurrence is also extremely rare. Due to the rarity of recurrent pouch volvulus, statistics on its prevalence remain unclear.

The aim of this report was to address the issue as to why such volvuli reoccur after a total proctocolectomy and IPAA surgery and to determine the best course of their management.

## Case presentation

**Figure 1:**
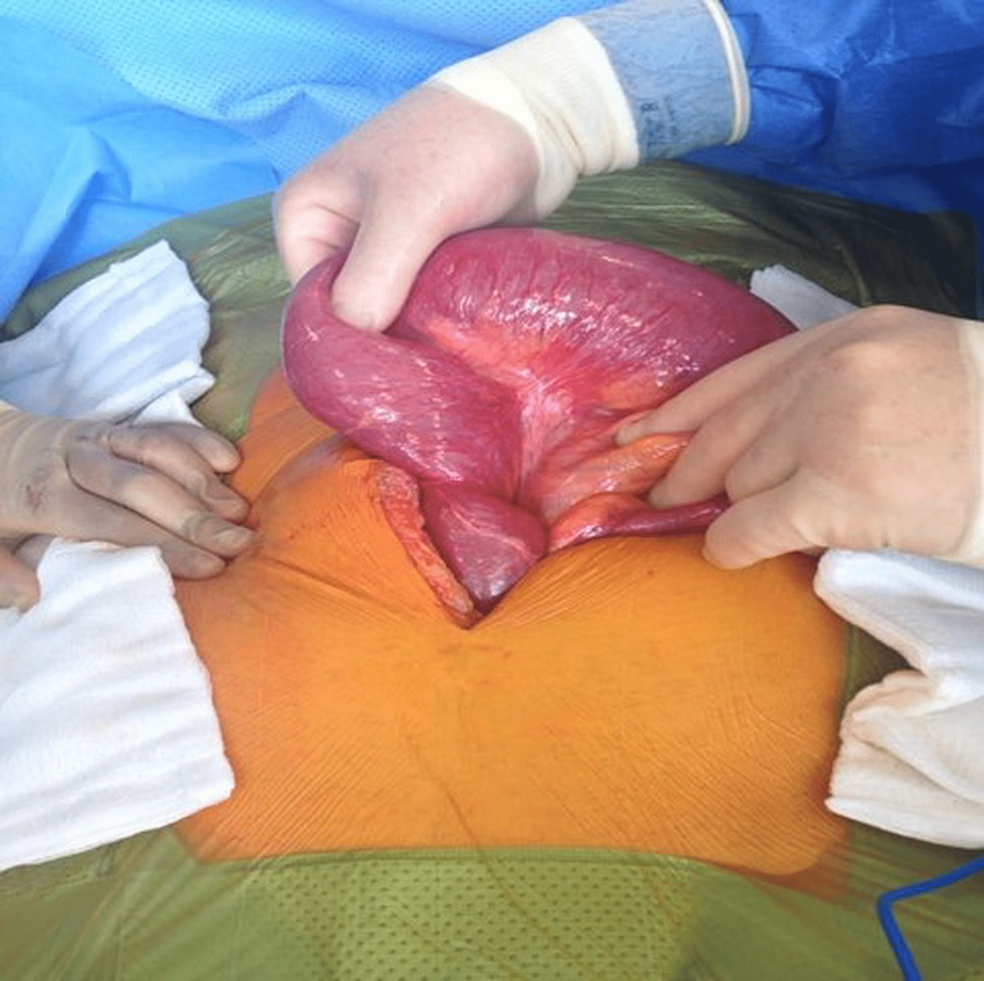
Exploratory laparotomy revealing distended pouch

**Figure 2:**
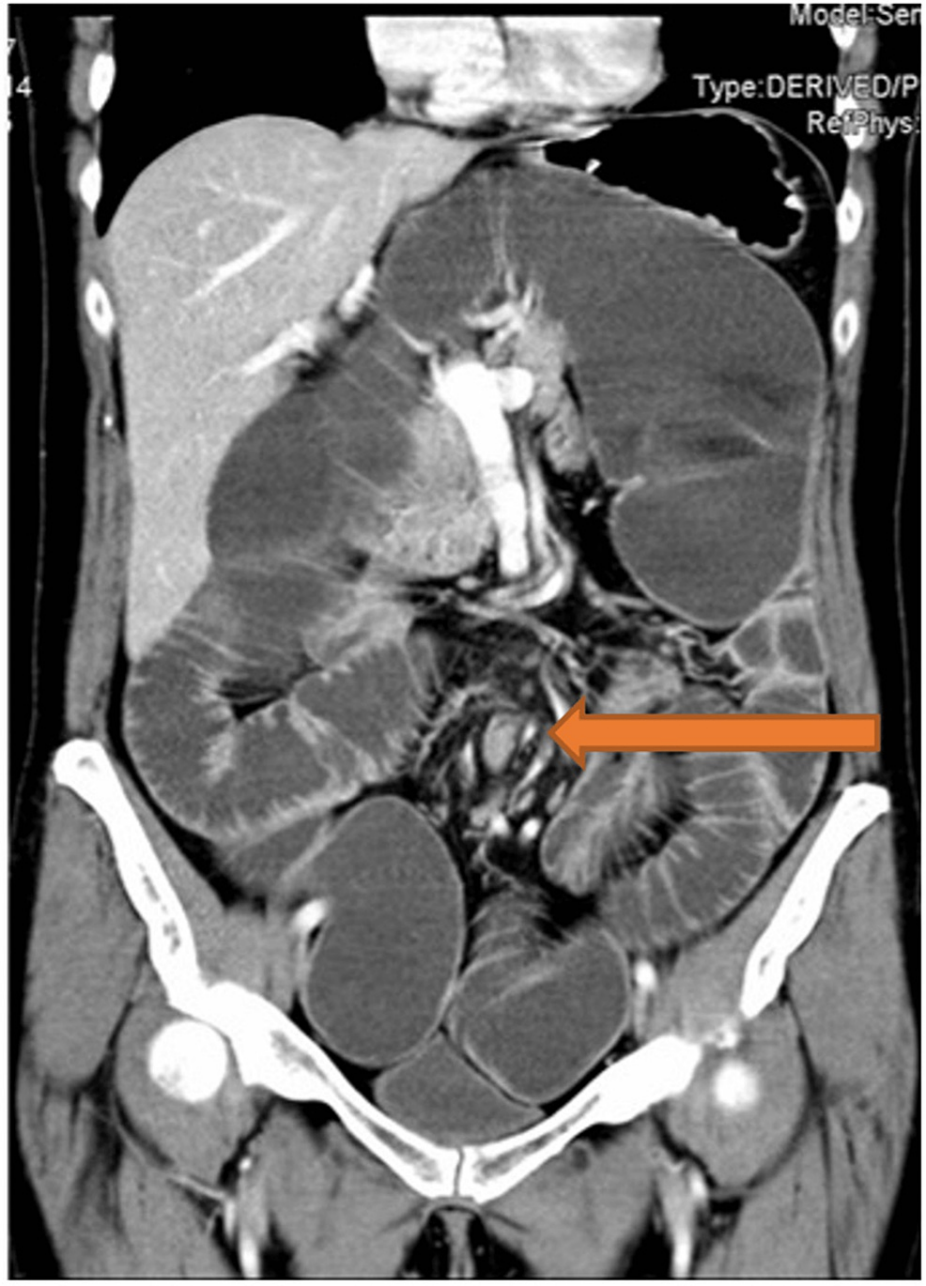
CT scan demonstrating the "whirlpool" sign of the mesentery (arrow) seen in pouch volvulus CT: computed tomography

A 57-year-old female initially presented to the colorectal surgery team in 1990 with refractory ulcerative colitis (UC). Prior to her presentation, she had undergone intensive medical treatment for UC; however, she was referred by gastroenterology due to the failure to respond to this treatment. Upon presentation, her vital signs were normal, but she had abdominal distention, central abdominal pain (without guarding), absolute constipation (last 24 hours), emesis, and was apyretic. There was no family history of any significance. The UC was debilitating in her everyday life as she had to pass stool five to six times daily. At the time, differential diagnoses included a *Clostridioides difficile* colitis infection and cytomegalovirus (CMV) colitis; however, both were ruled out. Crohn’s disease was also considered, but the patient’s clinical picture was more suggestive of UC. The decision was made for her to undergo a total proctocolectomy and an IPAA surgery. The outcome of this surgery was successful with no further complications until 15 years later (2005) when the patient presented to a hospital in Galway complaining of intermittent bouts of obstruction. At that time, a decompression endoscopy was performed along with an exploratory laparotomy (Figure 1); a dusky mucosa and "spokes of a wheel" presentation were discovered; however, no adhesions or necrosis were found. This decompression was done twice more and each time the patient presented with similar symptoms of obstruction. Ultimately, that same year, she was referred to the same surgeon who had performed the IPAA in 1990. Upon examination, her abdomen was soft and very distended, with tinkling bowel sounds. Her laboratory results showed elevated creatinine levels (115 µmol/l) with otherwise unremarkable results. Imaging was conducted and a CT scan revealed a pouch volvulus (Figure 2) in the ileal portion of the small bowel.

**Figure 3:**
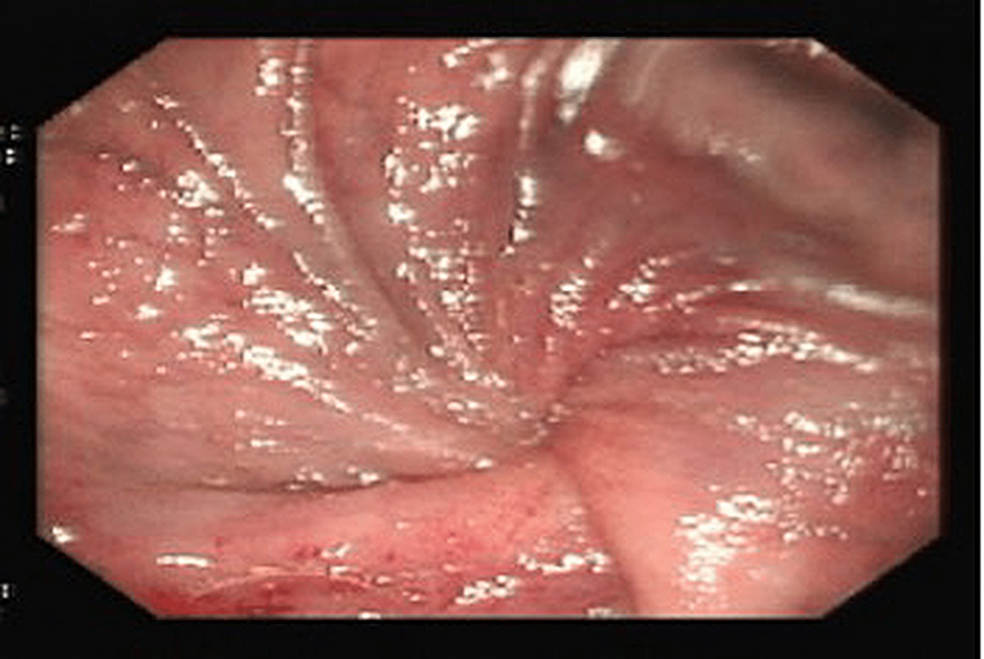
Flexible pouchoscopy revealing a "spokes of a wheel" pattern suggestive of volvulus

A further decompression endoscopy was performed, which revealed a dusky mucosa and "spokes of a wheel" presentation, similar to the first decompression she had undergone (Figure 3). The patient received a peripherally inserted central catheter and total parenteral nutrition thereafter but failed to settle after 48 hours. Due to the increased risk of perforation with every decompression, an ileectomy (50 cm) and enteropexy were performed via laparotomy. Their effect lasted only four months when the patient presented again with a remittent pouch volvulus. Ultimately, the decision was made to perform a loop ileostomy, and the patient, to date, is alive and doing well with this permanent ileostomy.

## Discussion

For individuals suffering from severe refractory UC who fail to respond to medical treatment, the gold-standard surgical treatment is a total proctocolectomy and IPAA surgery [2]. In this procedure, an ileal reservoir (J-pouch) is created, allowing for faecal continence at the ileoanal anastomotic junction. Typically, this pouch functions well and patients report good quality of life subsequently [3,4]. However, this procedure is associated with certain septic complications such as anastomotic leaks, pelvic or perianal abscesses, and pouch fistulas. Non-septic complications such as strictures, pouchitis, pouch prolapse, and volvulus may also occur [5]. Not only did the current patient present with the rare complication of volvulus, but she also presented with this complication on four further occasions, and hence simple decompressions were ineffective. To our knowledge, only seven papers have been published on recurrent pouch volvulus following an IPAA surgery, and hence key findings from this case raise the following question: after a total proctocolectomy and IPAA surgery for UC, why do such volvuli reoccur and what is the correct course of treatment for these?

Imaging, specifically CT, is a necessary component to diagnose pouch volvulus. CT scans can display evidence that is characteristic of volvulus, such as a whirl sign and air-fluid levels [6], as seen with the current patient (Figure 2). A whirl sign is indicative of a portion of the bowel looping around the axis of its mesentery [7]; therefore, an emergency decompression is often done as ischaemia and necrosis may occur. Ultrasound may be used to identify obstruction due to volvulus in pediatric patients, while X-ray may appear normal or show nonspecific bowel dilation [8,9].

To our knowledge, there is a dearth of case reports on patients with recurrent pouch volvulus after undergoing a total proctocolectomy and IPAA surgery. After an extensive PubMed search, we found only seven other cases that presented with recurrent pouch volvulus [10-16]. In five out of these seven cases, the initial volvulus was treated by decompression and a unilateral pouch pexy; however, this pexy did not suffice as the patient re-presented with a further volvulus [10,11,14-16]. At this point, the surgical teams had different approaches toward treating this recurrent volvulus: the treatment included performing a bilateral pouch pexy [11,14], creation of a new pouch [10], robotic ventral graft pexy [16], and using electrocautery to promote pouch adhesions [15]. Bilateral pouch pexy was done in two cases, with one of these cases [11] suggesting that a bilateral pouch pexy with multifilament sutures promotes adhesions, thereby further stabilising the pouch in the sacral hollow. Furthermore, the authors of this paper examined the position of the mesentery at the IPAA and whether this predisposes the pouch to volvulus [11]. Most often, IPAAs were constructed with the mesentery positioned anteriorly; however, some were also positioned posteriorly or on the right side. All patients presenting with recurrent volvulus were from the anterior group. Thus, the authors suspect that patients with an anteriorly positioned mesentery may be more prone to volvulus in the long term.

Another case study of note, by Perotti et al., performed electrocautery of the sacral hollow via laparoscopy to promote adhesion of the pouch [15]. The patient’s primary volvulus was treated by endoscopic decompression and a unilateral pexy after which she recovered uneventfully. The patient was said to have "a free-floating pouch without any adhesions between the pouch and the sacral hollow" [15], an observation also reported by all of the seven cases that we found in the literature [10-16]. One year later, the patient was diagnosed with a further volvulus for which she underwent a pouchoscopy and exploratory laparoscopy. All previous enteropexy sutures had pulled through and once again, the sacral hollow was peritonealised without any adhesions to the pouch. This time, however, electrocautery was done on the sacral hollow, thus promoting fibrosis and adhesions. Furthermore, a synthetic absorbable mesh was placed between the pouch and the sacral hollow, as the patient had suffered concurrently from an internal hernia beneath the mesentery of the J-pouch and the pre-sacral hollow. The patient recovered uneventfully with no further volvuli.

Traditionally, IPAA procedures were performed in an open fashion; however, with advances in medical technology, a minimally invasive approach using laparoscopy is now preferred, which has made recovery time quicker and the likelihood of adhesions lower [17,18]. Adhesions are considered unfavourable as these can lead to a higher chance of postoperative complications; however, a recent paper by Dionigi et al. [19] suggests that this decrease in adhesions through using laparoscopy, in fact, predisposes the pouch and its proximal small bowel to volvulus. They examined a series of IPAA surgeries performed over 40 years by multiple surgeons and found a low incidence of pouch volvulus: in total, only six out of 5760 patients developed a volvulus, all of which occurred after the introduction of minimally invasive surgery for IPAA procedures in 2012. Although laparoscopy was in use before 2012, the pelvic procedure of this operation was usually done via a Pfannenstiel incision, enabling sufficient visualisation to perform the anastomosis. As the skill required for using minimally invasive technology became more widely prevalent, a fully laparoscopic approach was, and still is, preferred. A previous retrospective study suggested that laparoscopic surgery is associated with fewer intraperitoneal adhesions due to the decreased surgical trauma [20]; however, following a pouch surgery, the formation of adhesions between the sacrum and the pouch, not the mesentery, may prove useful in stabilising the pouch within the pelvic cavity. These suggestions may be supported by Geers et al. and Perotti et al. [11,15], as both teams used means to promote adhesions between the pouch and the sacral hollow. 

Lastly, Itabashi et al. [12] suggested other factors that may predispose individuals to pouch volvulus, including the role of a prophylactic pouch pexy and the configuration of the pouch (the choice of J, S, or W pouch thus affecting pouch volume). Finally, the tension placed on the small bowel mesentery during manipulation upon creation of the IPAA may also contribute to the occurrence of volvulus.

We propose several recommendations for the management of pouch volvulus in the future, which include conducting more research on the prevalence of volvulus and underlying causes as to why it occurs, including genetic and environmental predisposition, as well as determining the best course of treatment upon patient presentation. Despite the rarity of pouch volvulus after IPAA surgery, recurrent volvuli can be extremely debilitating in everyday life, and hence an approach that provides certainty in terms of success needs to be identified. Lastly, more research should be done on the effects of promoting adhesions within the sacral hollow as a natural way of stabilising the pouch. Because most papers report a "free-floating pouch", natural stabilisation through adhesions in the initial IPAA surgery may drastically reduce the likelihood of pouch volvulus. It is important to note that these are only recommendations for the future and actual developments may vary from our proposals. The management of pouch volvulus will continue to evolve as new research and technologies emerge.

## Conclusions

We presented a case of recurrent pouch volvulus. Pouch volvulus is a rare but serious condition that can lead to significant morbidity and mortality if left untreated. The recurrence of this phenomenon is extremely rare and poorly understood. However, prompt diagnosis and management are essential to prevent serious complications. Further research is needed to better understand the epidemiology and pathophysiology of pouch volvulus and its recurrence, in order to develop improved diagnostic and therapeutic strategies.
